# Effectiveness of C1-INH therapy in angiotensin converting enzyme inhibitor induced angioedema

**DOI:** 10.1186/s13223-021-00521-w

**Published:** 2021-02-15

**Authors:** Uliana Kovaltchouk, Boyang Zhang, Vipul Jain, Chrystyna Kalicinsky

**Affiliations:** 1 Internal medicine, University of Manitoba, Winnipeg, Canada; 2 Max Rady College of Medicine, University of Manitoba, Winnipeg, Canada; 3 Clinal Immunology and Allergy, McMaster University, Hamilton, Canada; 4 Allergy and Immunology, University of Manitoba, Winnipeg, Canada

## Abstract

**Introduction:**

Angiotensin Converting Enzyme Inhibitors (ACEI) are a common cause of Emergency Room presentation for angioedema. Although no treatment guidelines exist, C1 esterase inhibitor concentrate (C1-INH) is used on an off label basis for management of ACEI acquired angioedema (ACEI AAE).

**Objective:**

To evaluate the efficacy of C1-INH in management of ACEI AAE at our local centers.

**Results:**

Nine patients, from 3 academic sites, were identified through Allergy Service consultation data and records from Diagnostic Services Manitoba, Canada from 2010–2020. The majority of the patients (n = 8/9) required endotracheal intubation prior to the initiation of C1-INH. Overall, approximately 56% of patients (n = 5/9) had resolution of angioedema ranging between 12 and 17 h, with a median time of 13.5 h, and no recurrence after the administration of C1-INH concentrate. One patient had transient symptom resolution in 14 h, however, recurrence of angioedema required re-intubation. The remainder of patients (n = 4/9), had resolution of angioedema between 22 and 72 h, with a median time of 33.75 h.

**Conclusion:**

Our findings demonstrate continued ambivalence of the efficacy and role of C1-INH concentrate in the treatment of ACEI AAE, secondary to multiple uncontrolled confounding factors. Further research into characterizing a subgroup of intubated patients in our study that responded to C1-INH concentrate needs to be completed.

## Background

Angiotensin Converting Enzyme Inhibitors (ACEI) are prescribed to over 40 million people for various indications, including heart failure, diabetes, chronic kidney disease, hypertension, and myocardial infarction [1]. Angioedema is a well-documented side effect of ACEI, affecting approximately 1% of patients, and is the leading cause of drug-induced angioedema emergency room visits [2–4]. The underlying physiological mechanism has been shown to be secondary to an excess of bradykinin and substance P, due to the inhibition of their degradation by ACE [5, 6]. There is no consensus on the best pharmacologic approach to managing patients with ACEI induced angioedema, nor is there any approved targeted treatment [7, 8]. Traditional pharmacological treatment modalities include antihistamines, steroids, epinephrine, all of which have been shown to be generally ineffective [9–11]. Without treatment, the edema typically resolves over 24–48 h, but, may take up to five days [12]. When symptoms of ACEI angioedema progress to threaten the airway, previous case reports and case series have described using bradykinin targeted treatments, including C1 esterase inhibitor (C1-INH) concentrate [11, 13–15]. The use of CI-INH concentrate) is controversial, given no phase 3 clinical studies have been performed to show its utility in managing ACEI angioedema. It currently remains an off-label treatment from its approved treatment indication for hereditary induced angioedema. Given the incidence of emergency room visits secondary to adverse reactions to ACEI, further assessment of the efficacy and utility of C1-INH concentrate in acute management is warranted.

## Methods

A retrospective chart review, from three academic hospitals in Winnipeg, Manitoba, was conducted examining all patients with ACEI induced angioedema, who received C1-INH treatment, between 2010 and 2020. This study was approved by the University of Manitoba Research Ethics Board. The inclusion criteria were as follows: patients must have been taking an ACEI at the time of developing angioedema, and have received C1-INH concentrate at one of the academic hospitals. The exclusion criteria was identifying angioedema caused by any other etiology. Data collected included patient demographics, medical indication for ACEI, alternative angioedema treatment prior to C1-INH concentrate administration, time of C1-INH concentrate administration, dose and number of doses of C1-INH concentrate administered, need for intubation, and time to symptom resolution after C1-INH concentrate administration.

## Results

Nine patients were identified through Allergy Service consultation data and records from Diagnostic Services Manitoba, Canada from 2010 to 2020. The majority of patients identified were female (n = 6/9), and of Caucasian ethnicity (n = 8/9), with the exception of one Filipino patient. Three patients received ACEi therapy with ramipril, two with perindopril, two with enalapril, one with lisinopril, and one with quinapril. Less than half of the patients analyzed were being treated with NSAIDs at the time of developing angioedema (n = 4/9). All patient suffered from hypertension, with five patients also having a concomitant diagnosis of diabetes. All patients had an acute angioedema attack of the upper airway or face, including lips, tongue, soft palate, and/or cheeks. Initial management of all patients was with methylprednisolone (dosed between 60-80 mg IV), IV diphenhydramine (dosed at 50 mg) and epinephrine (dosed between 0.1 mg IV or, 0.3–0.5 mg IM), either by paramedics, or emergency room physicians at the time of presentation. The majority of the patients (n = 7/9) required endotracheal intubation prior to the initiation of C1-INH concentrate for airway protection. The time to receiving C1-INH concentrate in the emergency room was highly variable, with the range being between 1 and 25 h (median 8.25 h). The first dose of C1-INH concentrate provided was 20 units/kg, with doses ranging from 1000 to 1500 IU. Three patients received an additional dose of C1-INH concentrate: one patient received an additional 500 IU, while the remaining two patients received 1000 IU. One patient was initially managed with one dose of C1-INH concentrate 1500 IU, however, went on 4 h later to receive one dose of Icabitant 30 mg subcutaneously, given minimal initial response to C1-INH concentrate treatment. This patient was extubated 26 h after receiving both C1-INH concentrate and Icabitant. Overall, approximately 44% of patients (n = 4/9) had resolution of angioedema ranging between 12 and 14 h, with a median time of 12.75 h, and no recurrence. One patient had transient symptom resolution in 14 h, however, recurrence of angioedema 24 h later, requiring re-intubation. The remainder of patients (n = 4/9), had resolution of angioedema between 22 and 72 h, with a median time of 33.75 h. Summary of these findings are found in Table 1.

**Table 1:** Summary of demographic findings and timing of C1-INH administration, dose, and time to angioedema resolution (marked by time to extubation)

Case	Age	Sex	ACEi	Diabetes	NSAIDs	Time from onset of symptoms to drug administration	Dose of Berinert	Time to Resolution of symptoms
1	80	F	Ramipril	No	No	6.5 h	1500 IU	12.5 h
2	57	F	Ramipril	No	No	22 h	1500 IU	12 h
3	67	F	Perindopril	Yes	Yes	9.5 h	1000 IU 500 IU	35 h
4	70	M	Perindopril	Yes	Yes	1 h	1500 IU	32.5 h
5	77	M	Quinapril	Yes	Yes	7 h	1500 IU 1000 IU	72 h
6	62	F	Lisinopril	No	No	5 h Recurrence of symptoms 24 h later→ 4 h	1500 IU 1000 IU	14 h 26 h
7	64	F	Enalapril	No	No	25 h	1000 IU	13 h
8	67	F	Ramipril	Yes	No	10 h	1500 IU	No response within 4 h → received 30 mg Icabitant → 22 h
9	70	M	Enalapril	Yes	No	11 h	1500 IU	14 h

## Discussion

The evidence supporting use of C1-INH concentrate, or Berinert, for ACEI AAE is limited in the current medical literature. Previous case and small case series reports have shown highly variable symptom response, no significant prevention of endotracheal intubation, and no reduction in the time spent in the intensive care unit [11, 13–15]. Despite our study not being designed as an interventional trial, our findings are unique from previous case series described, in that the majority of the patients analyzed were intubated for airway protection, prior to C1-INH concentrate treatment. Additionally, our findings of symptom resolution, which we have defined as time to extubation, were dichotomous, with approximately half of patients improving within 12 h, and the other half over 24 h. C1-INH functions through inactivating plasma kallikrein and factor XIIa, which raises the question of whether the dichotomy could be explained by variations in levels of both of these factors in patients admitted to the intensive care unit. Additionally, there was a trend that patients being treated with NSAIDs at the time of developing the angioedema, responded slower to the C1-INH treatment. This raises the question of whether there was a multifactorial etiology, or alternative explanation of the angioedema in these patients, given the mechanism of NSAID induced angioedema is through shunting of arachidonic acid towards the 5-lipoxygenase pathway, resulting in increased synthesis of cysteinyl leukotrienes [16]. Given the limited number of patients analyzed, a significant correlation between the time of administration of C1-INH concentrate, and subsequent time to symptom resolution, was not able to be made, in terms of specific patient characteristics. However, given that no adverse reactions to C1-INH concentrate were documented, it can be speculated that in a subgroup of intubated patients, C1-INH concentrate treatment does shorten the duration of symptoms, and leads to a reduction in time being intubated. Providing an additional dose of C1-INH concentrate after a period of no response from an initial dose, also, did not improve time to resolution of symptoms in two patients, with the times to symptom resolution being at 35 and 72 h. This suggests that if a response to C1-INH concentrate treatment will be seen, it will be with the first dose. Major limitations to this study revolve around the study being retrospective in nature. Our small sample size is reflective of the overall infrequent presentation of ACEI AAE. Factors such as angioedema that may have been secondary to NSAIDs or histamine mediated angioedema were not able to be accounted for asides from preliminary demographic screening (screening patients who on history were on an ACEI and developed angioedema without a clear alternative cause for their angioedema). The use of epinephrine, corticosteroids, and antihistamines prior to the administration of C1-INH concentrate are additional confounders in this study—however, the administration of all of these were done prior to intubation. Given no response to the above treatment, and the history being unrevealing, it was assumed that histaminergic or mast cell-mediated angioedema was not a significant confounding variable in this study.

## Conclusions

ACEI angioedema is a severe and potentially fatal side effect. As ACEI are still widely used worldwide for a variety of medical conditions, an optimal management strategy for patients experiencing acute ACEi angioedema is needed. To date, there is no specific pharmacological intervention that has been approved. This study adds to the current medical literature, as we have specifically investigated the efficacy of C1-INH concentrate administration in patients who have been intubated for airway protection from ACEi induced angioedema. Although our preliminary findings suggest that C1-INH concentrate administration may shorten the time spent in the intensive care unit in a subgroup of patients, this conclusion must be met with caution given multiple confounding variables. Ultimately, further research into characterizing this subgroup of patients needs to be completed.

## Data Availability

Data results reported in this manuscript are stored on a private, password, and virus-protected computer, and, are available on reasonable request. No hyperlinks to publicly archived datasets have been generated during the study.
